# Safety and efficacy of CyTisine for smoking cessation in a hOSPital context (CITOSP): study protocol for a prospective observational study

**DOI:** 10.3389/fpubh.2024.1350176

**Published:** 2024-09-30

**Authors:** Alessandro Torazzi, Erika Tedesco, Sofia Ceccato, Laura Santin, Simone Campagnari, Lorenzo Losso, Silvia Toldo, Rebecca Casari, Elena Arzenton, Paola Marini, Cristiano Chiamulera, Fabio Lugoboni

**Affiliations:** ^1^Department Diagnostic and Public Health, University of Verona, Verona, Italy; ^2^Department of Pharmacy, Integrated University Hospital of Verona, Verona, Italy; ^3^Unit of Addiction Medicine, Department of Internal Medicine, Integrated University Hospital of Verona, Policlinico “G.B. Rossi”, Verona, Italy

**Keywords:** cytisine, smoking cessation, tobacco addiction, hospital setting, safety, protocol, cost-effective

## Abstract

Tobacco addiction is the primary preventable factor contributing to global mortality, and nicotine is one of the substances with the greatest potential for addiction. With a strong affinity for the α4β2 subtype receptor, cytisine (CYT) functions as a partial agonist of the acetylcholine nicotinic cholinergic receptor. It counteracts the effects of nicotine without causing any withdrawal symptoms. These features, combined with its limited mild adverse effects and minimal drug–drug interactions, make cytisine a cost-effective treatment for smoking cessation. The current protocol describes a prospective observational study on the safety and efficacy of CYT administered to inpatient smokers of the Integrated University Hospital of Verona (IUHVR), Veneto (Italy). This is a monocentric, observational, and prospective study on both sex smokers over the age of 18 years admitted to the IUHVR who meet the criteria for recruitment and have given their consent. Eligible participants will be assigned to the CYT intervention based on the West dosing schedule and will be followed up for 12 months from treatment initiation. Evaluation of safety, efficacy, and compliance will be assessed at 7 and 25 days, with follow-up at 3, 6, and 12 months from the start of the treatment (quit day). During each visit, any adverse events or adverse reactions reported by patients following the intake of CYT will be evaluated. This study will contribute, for the first time, to the knowledge about the use of CYT for smoking cessation in a hospital setting.

## Introduction

Cigarette smoking stands as the primary avoidable factor responsible for diseases and premature mortality on a global scale. According to the World Health Organization (WHO), smoking causes the deaths of over eight million people annually (1, 2). A total of 1.18 billion individuals smoked cigarettes in 2020, with a global smoking prevalence of 6.5% among women and 32.6% among men, causing 7 million deaths (2).

Smoking is responsible not only for several neoplastic pathologies, such as lung cancer, but also for non-neoplastic diseases, among others chronic obstructive pulmonary disease (COPD) and cardiovascular diseases (3). In Italy, approximately 50% of smokers die prematurely because of smoking-related diseases, and data relating to the younger population are particularly alarming (4).

Nicotine, an alkaloid (1-methyl-2-43 [3-pyrodyl] pyrrolidine), is the predominant pharmacological substance implicated in tobacco addiction, and its effect is related to binding the cholinergic receptors, which are involved in the addiction mechanism due to dopamine release. Like all other pathologies, nicotine addiction is a clinical condition that requires medical care and must be treated using pharmacological and non-pharmacological strategies (1).

Pharmacological treatments for tobacco addiction act on the dopaminergic and cholinergic systems in order to prevent or reduce nicotine withdrawal symptoms (5). Nicotine substitutes (nicotine replacement therapy, NRT), bupropion, varenicline, and cytisine (CYT) have all demonstrated efficacy in mitigating nicotine withdrawal (6).

Varenicline functions as a partial agonist of the brain’s α4β2 subtype of the nicotinic acetylcholine receptor (nAChR), a receptor closely associated with nicotine dependence (7). Operating as a partial agonist, its mechanism involves both agonistic properties, capable of moderating symptoms associated with nicotine withdrawal, and antagonistic properties, which can reduce the pleasurable effects of nicotine (8, 9).

CYT is a quinolizidine alkaloid of natural origin extracted mainly from the seeds of plants that are part of the *Leguminosae* family, including *Cytisus Laburnum,* and, such as varenicline, is a partial agonist of the nAChR (10–13).

CYT has the ability to act as a partial agonist, and its strong affinity for the subtype receptor α4β2 counteracts the addictive properties of nicotine, reducing withdrawal symptoms (14, 15). After nAChR binding with CYT, the restrained release of dopamine could block the effect of nicotine from tobacco on the receptor (16, 17). In particular, several studies have indicated that in the context of nicotine addiction, the administration of CYT may have a dual impact. On one hand, it could modestly elevate dopamine levels within the mesolimbic system, reaching approximately half the levels induced by nicotine. On the other hand, CYT might mitigate withdrawal symptoms, reducing the sudden spike in dopamine levels linked to the rapid absorption of nicotine typically associated with cigarette smoking (18, 19).

The first clinical study on CYT was carried out in 1965, and in the following 10 years, different studies were conducted in many European countries such as Bulgaria, Poland, Germany, and Russia (20, 21). The findings achieved by these trials kicked off the widespread use of the CYT commercial product branded TABEX®, which was first marketed in Bulgaria in 1964 and then became widely available in former socialist economy (FSE) countries, including Poland (22). After the establishment of the European Union (EU), some FSE countries joined it, and TABEX® was withdrawn from some of them. However, TABEX® is still prescribed in Poland (7, 23). In Italy, CYT has been available since 2015 and is currently classified as an active pharmaceutical ingredient (API). Since no CYT-based pharmaceutical products are commercially available in Italy, CYT is imported in the form of raw material, which can be purchased by territorial and hospital pharmacies (HPs) for galenic capsule production. According to Italian law, CYT galenic capsules can be sold and dispensed to patients only under medical prescription.

Due to its natural and traditional origin, CYT’s development is characterized by an unusual history, and standard preclinical research to establish the optimal dosage, which would normally precede randomized clinical trials, was not carried out. To assess CYT pharmacokinetics, many studies were conducted in animal models; however, pharmacokinetic data in humans are limited (24). Nevertheless, findings gathered from these studies revealed that the half-life of CYT is approximately 4.8 h, with 95% being eliminated through urine unmodified (25, 26). A further important aspect of CYT’s pharmacokinetics is that there is no hepatic metabolism, and it is possible to exclude drug–drug interactions (27). The West dosing schedule (1.5 to 9 mg/day for 25 days) has been licensed for CYT in several countries, and it was used in an observational study in which participants treated with CYT had significantly high quit rates to suggest efficacy (7).

According to West et al. in 2011, CYT treatment is generally well tolerated. In fact, in this clinical trial, CYT is not associated with an increased risk of adverse events compared to placebo, although gastrointestinal symptoms are more frequent than placebo. The most frequent adverse reactions reported are abnormal dreams, insomnia, changes in taste, dry mouth and throat, decreased appetite, and, less frequently, nausea. Some patients have experienced headaches and irritability on the initial day of treatment. It is also noted that elevated doses of CYT can lead to dizziness and muscle weakness. However, reducing the dosage has been shown to alleviate these side effects. Importantly, CYT does not induce psychophysical alterations, making it suitable for individuals who drive vehicles or operate machinery (28, 29).

In Italy, the use of CYT is promoted by the Veneto regional program “Coordination and development of the Veneto system of tobacco treatment” for active smokers, especially those who smoke more than 15 cigarettes per day and are both available and motivated to quit smoking.

Since 2016, the galenic laboratory of HPs in the Integrated University Hospital of Verona (IUHVR), Veneto (Italy), has provided 1.5 mg CYT galenic capsules for in-hospital treatment for patients.

One of the advantages of introducing CYT in a hospital setting is to decrease costs related to NRT during hospitalization. In addition, the low cost of CYT could encourage its use after discharge, thus minimizing the risk of relapse and encouraging therapeutic continuity. It is important to emphasize that CYT is, by far, the cheapest anti-smoking option, compared to other pharmacological interventions, making it particularly appreciated, especially by heavy smokers, who generally require higher therapeutic doses and longer treatment periods (19). Therefore, CYT may be an attractive choice for smokers due to its lower cost in comparison to other pharmacotherapies.

Although actual knowledge about CYT’s pharmacokinetics suggests few drug interactions, the administration in hospitalized patients, who often present co-morbidity and polypharmacotherapy, has not yet been investigated. This protocol describes a prospective observational study to investigate the safety of CYT prescribed to a sample of smoking patients in a hospital setting.

The findings from this study will enhance our understanding of CYT management, promoting smoking cessation intervention through the use of an effective and safe substance as part of an integrated cessation and relapse prevention intervention.

## Methods

### Objectives of the study

The primary objective of the study is to assess the safety of CYT treatment in hospitalized smokers admitted to IUHVR.

The secondary objectives of this study are to assess patient’s compliance with CYT treatment and the efficacy of CYT treatment.

### Study design

This study is a monocentric, observational prospective investigation involving smoking patients admitted to IUHVR and treated with CYT for smoking cessation.

### Clinical endpoints

The primary endpoint of the study is to assess the safety of CYT adverse events (AEs) or adverse drug reactions (ADRs) reported by participants, which will be collected.

AEs are defined by the Italian Legislative Decree 211/2003 as “any harmful clinical event that occurs in a patient or in a person involved in a clinical trial that has been given a medicinal/device product and does not necessarily have a causal relationship with this treatment.” Indeed, ADRs are defined as follows: “all untoward and unintended responses to an investigational medicinal product related to any dose administered.” The definition also covers “medication errors and uses outside what is foreseen in the protocol, including misuse and abuse of the product” (Directive 2001/83/EC, Ministerial Decree 30 April 2015-GU No. 143).

For the European Regulation (Directive 2001/83/EC), serious adverse events (SAEs) or serious adverse reactions (SARs) are defined as “any unwanted medical event or device failure that results in the following outcomes: is fatal (causes death), is life-threatening (an event in which the patient was at risk of death at the time of the event), requests hospitalization or extension of hospitalization in progress, determines persistent or significant degree disability or incapacity, corresponds to a congenital anomaly/birth defect, is medically significant, or requires intervention to prevent one of the other outcomes listed above.”

If an AE occurs, the physician, supported by the clinical monitor (CM), will evaluate the event/treatment correlation to determine if an AE could be an ADR. At each visit or follow-up, AEs and ADRs reported by participants will be recorded by the CM in the CRF. In case of an ADR, the physician or the CM will fill out the online reporting form on the AIFA website (https://servizionline.aifa.gov.it/schedasegnalazioni/#/). The textual descriptions will be summarized in the report forms and will be coded according to standard terms in the Medical Dictionary for Regulatory Activities (MedDRA).

The secondary endpoints of the study are to assess the efficacy and compliance of CYT treatment. Self-reported 7-day and 15-day point prevalence abstinence (PPA) will be assessed at each study timepoint. A relapse will occur when patients smoke 7 or more cigarettes for 7 consecutive days or 2 consecutive weeks.

Biochemically verified abstinence will be evaluated using exhaled carbon monoxide (CO) measurement during in-person visits. A relapse is considered when CO value ≥9 ppm (non-smoker value <4.5 ppm) (30, 31).

To assess CYT treatment compliance, patients will be provided a study diary in which capsule intake or self-reported treatment adherence can be reported.

### Setting and participants

Participants over 18 years of age will be recruited from IUHVR. During the baseline visit, participants will be assessed and evaluated for eligibility, and informed written consent will be signed. Eligible participants will be allocated to the CYT treatment and will be followed up for an entire year from treatment initiation.

#### Inclusion criteria

The inclusion criteria are: adults ≥18 years old admitted to IUHVR participating wards with a history of smoking and interested in quitting. They must also be willing to utilize CYT pharmacotherapy, be available for a 12-month follow-up after treatment begins, and be able to provide written informed consent.

#### Exclusion criteria

Patients who do not fit all of the requirements mentioned above, those who have any contraindications for the use of CYT, such as reported hypersensitivity to CYT, pheochromocytoma, malignant hypertension, unstable angina, will be excluded from the study, as will women who are breastfeeding, pregnant or likely to become pregnant during CYT treatment and are not willing to use an effective form of contraception.

Until the baseline visit, the use of other forms of nicotine delivery, such as electronic cigarettes, in combination with industrial cigarettes, will not be an exclusion criterion. However, the use of nicotine-containing products as smoking cessation medications (e.g., NRTs) starting from the QD will cause the exclusion of participants from the study, while the use of co-adjuvants for smoking cessation (e.g., electronic cigarettes without nicotine, Nirdosh®) will be allowed, according to clinical practice, and will be recorded in eCRF at each visit.

Because CYT is eliminated through urine unmodified, patients with chronic kidney failure will be closely monitored but not excluded from the trial. Patients in stage IV or V, with glomerular filtration rate (GFR) < 29 mL/min, will receive CYT treatment with halved dosage; patients in stage III, with GFR between 30 and 59 mL/min, will receive standard CYT treatment, but close patient monitoring will be performed.

### Participant recruitment

Participating ward staff will be used to screen for and determine eligible participants, by physicians trained in the study by the principal investigator (PI) or sub-investigators (Sub-I), such as addiction medicine physicians (AMPs) or CM. Once potential participants are identified, ward staff will refer all current smokers interested in quitting smoking to the CM. Each patient’s eligibility for the study will be determined by the AMPs in collaboration with the CM, taking into account the patient’s current medical status and any contraindications to the usage of CYT.

The CM will then approach eligible patients, describe the CITOSP trial’s details to each potential participant, and answer any questions. If patients are willing to participate, prior to completing the baseline interview, written informed consent will be obtained.

Each participant will receive an anonymized study code at the baseline interview, and baseline data will be collected. This data will include general demographics such as age, gender, highest level of education, and current job position. Detailed medical histories will be considered, including medical conditions, current and remote anamnesis, and concomitant medications. Additionally, information on the participant’s smoking habits will be reviewed, such as age at smoking onset, cigarettes smoked per day, and previous attempts at quitting. Furthermore, the level of cigarette dependence assessed using the Fagerström Test for Cigarette Dependence (FTND) Questionnaire will be collected. The FTND is a validated and widely used questionnaire consisting of six items that measure the degree of nicotine dependence in tobacco smokers. The FTND yields a final total score ranging from 0 to 10. The patient’s physical nicotine dependence is closely related to the FTDN score. Scores from 0 to 2 indicate very low nicotine addiction; scores 3 and 4 indicate low nicotine addiction; a score of 5 indicates an average nicotine addiction; scores 6 and 7 indicate high nicotine addiction; and scores from 8 to 10 indicate very high addiction (32).

The decision concerning the patient’s inclusion in the study will ultimately be made by the AMPs and the CM based on the predefined inclusion and exclusion criteria. Any participant who meets the exclusion criteria will not be recruited. Following the collection of baseline data, the AMPs and the CM will then give CYT, prepared by IUHVR’s HPs, to the participants and provide detailed counseling. In case of discharge, CYT will be provided to the patients to continue the therapy at home. CYT will be provided throughout the treatment duration according to the West dosing schedule (25 days, 101 capsules). In the case of patients with chronic kidney failure (stage IV or V), a reduced dosage schedule will be followed, providing 57 capsules.

On the first day of therapy, all participants will be instructed to abstain from smoking and to quit completely (quit day, QD).

Instructions about the West dosage schedule, common CYT ADRs, and emergency contact information will be given to participants by the CM. Finally, a study diary will be provided to support participants to help ensure proper intake of CYT.

### Visits and follow-ups

**Figure 1:**
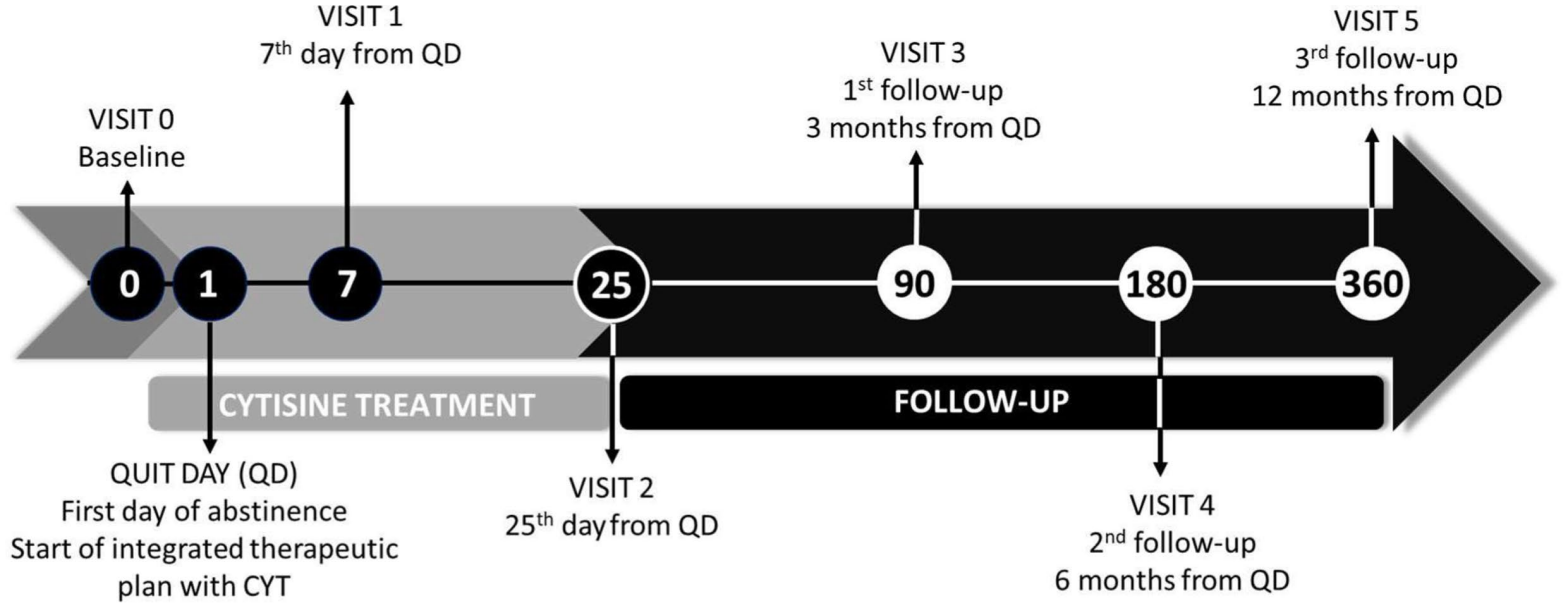
Study protocol timeline. QD, quit day.

All participants will be followed up for a period of 12 months after treatment initiation (Figure 1).

After the baseline visit, two visits (visit 1 at day 7 and visit 2 at the end of treatment, day 25) and three follow-ups (visit 3 at 3 months, visit 4 at 6 months, and visit 5 at 12 months) will be conducted (see Table 1). Visits 1, 2, and 3 will be conducted face-to-face. The last two visits (visits 4 and 5) will be conducted via telephone.

**Table 1:** Protocol timeline procedure of visits and follow-up for patients enrolled in the CITOSP trial.

Procedure	Visit 0	QD*	Visit 1	Visit 2^§^	Visit 3	Visit 4	Visit 5
Informed consent	√						
Inclusion/exclusion criteria	√						
Demographic characteristics	√						
Clinical history	√						
Smoking history and status	√						
Cytisine treatment		√	√	√			
Adverse events			√	√	√	√	√
Adverse drug reactions			√	√	√	√	√
Self-reported abstinence (PPA)			√	√	√	√	√
Biochemically verified abstinence (exhaled CO)			√	√^#^	√^#^		

QD, quit day.

PPA, point prevalence abstinence.

CO, carbon monoxide.

^#^Only if the visit is in person.

*First day of treatment. ^§^Stop treatment.

Each control visit resulted in the collection of the following records:

Visit 0 (baseline visit): informed consent dated and signed, participant identification code, and socio-demographic characteristics of the participant (age, sex, education, and occupation). Smoking history: age at which cigarettes were first smoked and efforts to quit smoking (number of times, date of last attempt, and use of some drugs to quit smoking). Smoking Status: average amount of cigarettes smoked in the last month, average cigarettes for the year, the score obtained in the FTND test, and use of other forms of nicotine delivery (for example, e-cigarettes). Clinical history: pathologies and ongoing pharmacological treatments. Integrated therapeutic: This statement outlines the non-pharmacological approaches used in the study to help patients, including counseling, cognitive-behavioral education, lifestyle changes, and the possible use of smoking cessation tools.

QD (first day of abstinence): start of integrated therapeutic plan with CYT.

Visit 1 (7^th^ from QD): continuation of the integrated therapeutic plan with CYT, evaluation of AEs, evaluation of ADRs, evaluation of PPA, number of cigarettes smoked (in case of quit failure), and exhaled CO.

Visit 2 (25^th^ day from QD): end of treatment with CYT, evaluation of AEs, evaluation of ADRs, evaluation of PPA, and exhaled CO.

Visit 3 (first follow-up, 3 months from QD): evaluation of AEs, evaluation of ADRs, evaluation of PPA, number of cigarettes smoked (in the case of failure to quit), and exhaled CO.

Visit 4 (second follow-up, 6 months from QD): evaluation of AEs, evaluation of ADRs, evaluation of PPA, and number of cigarettes smoked (in the case of failure to quit).

Visit 5 (third follow-up, 12 months from QD): evaluation of AEs, evaluation of ADRs, evaluation of PPA, and number of cigarettes smoked (in the case of failure to quit).

### Cytisine dosing schedule

The West dosing schedule consisted of six 1.5 mg capsules per day (one capsule every 2 h) for 3 days (days 1 to 3), five capsules per day for 9 days (days 4 to 12), four capsules per day for 4 days (days 13 to 16), three capsules per day for 4 days (days 17 to 20), and two capsules per day for the final 5 days (days 21 to 25) (see Table 2). The target quit date was scheduled for the first day of treatment (day 1) (7).

**Table 2:** West dosing schedule (7).

Day of treatment	N° of capsules/die	Frequency of administration
Days 1–3	6	1 capsule every 2 h
Days 4–12	5	1 capsule every 2.5 h
Days 13–16	4	1 capsule every 3 h
Days 17–20	3	1 capsule every 4 h
Days 21–25	2	1 capsule every 6 h

In the case of patients with stage IV or V chronic kidney failure, the CYT dosing schedule consisted of three 1.5-mg capsules per day for 12 days (days 1 to 12), two capsules per day for 8 days (days 13 to 20), and finally one capsule per day for the last 5 days (days 21 to 25) (see Table 3).

**Table 3:** Cytisine dosing schedule adapted to patients in stage IV or V chronic kidney failure.

Day of treatment	N° of capsules/die	Frequency of administration
Days 1–12	3	1 capsule every 4 h
Days 13–20	2	1 capsule every 6 h
Days 21–25	1	1 capsule every 12 h

Concomitant medication use during CYT treatment will be allowed, evaluated, and documented at each visit, and any possible interactions with CYT will be verified.

### Carbon monoxide measurement

The exhaled CO concentration will be measured using the piCOTM Smokerlyzer® (Bedfont Instruments; Kent, United Kingdom), a non-invasive method for assessing the smoker status. It has been reported that the concentration of CO is closely related to the concentration of carboxyhemoglobin in smokers’ and non-smokers’ blood. The piCOTM Smokerlyzer® measures CO levels in the air in parts per million (ppm) based on the conversion of CO to carbon dioxide (CO_2_) on a catalytically active electrode (33).

Scores from 0 ppm to 6 ppm confirm that patients have not smoked cigarettes in previous days before the visit, scores 7 ppm and 8 ppm indicate that patients are light smokers; scores greater or equal to 9 ppm confirm that patients are heavy smokers.

### Sample size and statistical analysis

Assuming an overall incidence of all AEs and ADRs equal to 20% as reported by Hajek et al. (28), 264 subjects will be needed to produce a bilateral confidence interval of 95% with an amplitude of 10%. This number will be increased to 300 subjects to compensate for a potential loss of 14% of subjects during the 12-month follow-up. To recruit 300 subjects, it will take 36 months. The total duration of the study will be 4 years.

All variables recorded in the study will be presented using the plus descriptive statistic appropriate to their nature. Continuous variables will be presented with mean and standard deviation with median and interquartile distance based on their distribution; ordinal and categorical variables will be presented with frequency and percentage.

To assess the safety of treatment with CYT, the incidence of AEs and ADRs will be evaluated, with respective 95% confidence intervals (CIs) up to the end of treatment (day 25) and up to 12 months from the start of the treatment (quit day). The percentage of subjects will be calculated to assess the compliance of patients with CYT treatment who respected their dosage schedule with the respective 95% CI.

To evaluate the efficacy of CYT treatment, the percentage of subjects who reported abstinence in the previous 7 days and 15 days (7-day PPA and 15-day PPA) before each study timepoint will be calculated. For in-person visits and follow-ups, the PPA will be biochemically verified through exhaled CO measurement.

The efficacy will also be studied at a multivariate level to assess whether the positive/negative outcome of the treatment is associated with certain demographic and/or clinical factors. For this type of analysis, they will construct logistic regression models by setting the (yes/no) relapse evaluated as dependent variables according to the reported and objective efficacy criteria at the end of treatment (25^th^ day from the start of treatment) and 12 months from the start of treatment. The independent variables will be chosen based on the assumptions of experimenters on possible predictors of efficacy, including the result of the FTND, the ward, the primary cause of admission, sex, age, education, occupation, age in which the person started smoking, and number of previous smoking cessation attempts.

### Ethics and data security

The trial will be conducted in compliance with the protocol and the legal standard required for clinical trials in EU and Italy: The principles of Good Clinical Practice (ICH Harmonized Tripartite Guidelines for Good Clinical Practice 1996), Directive 91/507/EEC, The Rules Governing Medicinal Products in the European Community, Legislative Decree n.211 of 24 June 2003, Legislative Decree n.200 6 November 2007, Ministerial Decree 21 December 2007, and AIFA Determination 20 March 2008.

All essential clinical documents will be maintained to demonstrate the validity of the study and the integrity of the collected data. The promoter of this study (IUHVR) will process the personal data collected, exclusively for the purpose of carrying out the study and for pharmacovigilance purposes.

The study approval has been obtained from the local Ethics Committees of IUHVR. Ethics approval for the trial was obtained on 21/09/2021.

At the time of recruiting, each participant will be required to provide written informed consent. At the IUHVR, any information that is personally identifiable will be safely kept in secured filing cabinets and/or password-protected computers at the IUHVR. In particular, to ensure the secrecy of the data as well as to avoid the manipulation and loss of the data, the following precautionary measures have been adopted: access data is reserved only for authorized members (PI and Sub-I); the network is protected by a firewall; Internet connection is encrypted with a digital certificate (SSL technology); the database is located on a server, protected by a password that is changed periodically and the access to the database is password protected and is accessible only to the persons responsible for authorized members. Finally, periodic back-ups are performed.

Before acquiring the data, investigators undertake to provide appropriate information to each patient about the nature, purpose, results, consequences, and risks of the study prior to their participation in the study. Before enrolling the patient, the PI or Sub-I will obtain the patient’s informed consent to participate in the study and collect their personal treatment data. After removing identifying information, the collected data will be entered into the Research Electronic Data Capture (REDCap) electronic Case Report Form (eCRF). The research team will submit study findings to peer-reviewed journals.

## Discussion

Focus on patient safety during the treatment process is a crucial aspect of the effective operation of healthcare systems globally. Undesirable pharmacological interactions have the potential to elevate a drug’s toxicity or diminish its effectiveness (34). Smoking is a cross-cutting problem in the medical field, and various specialists are seeking a valid, safe, effective, handy, and low-cost treatment for the cessation of cigarette smoking, including for inpatients. In fact, the majority of hospitalized smokers present co-morbid conditions, such as smoking-related diseases. For this population, smoking cessation is often mandatory, and physicians need safe and effective treatment options to reduce withdrawal symptoms and prevent relapse after discharge.

During hospitalization, treating withdrawal symptoms in smokers is essential. The ideal pharmacological treatment for smoking cessation in hospitalized patients with co-morbid conditions, who are often on polypharmacotherapy, should be handy, effective, safe, and well-tolerated in order to prevent further compromising their health. The following are predisposing factors for drug-related AE occurrence: advanced age, multiple morbidity, and polypharmacotherapy. It is important to consider that in wealthy countries, approximately 30–40% of those over 65 years of age take 5 or more drugs, and 12% use 10 or more medications (34).

Evidence about CYT’s pharmacokinetic profile suggests no or few drug interactions. For this reason, CYT could be considered a safe option for hospitalized smokers, who are often assuming concomitant therapies. Despite this, there is a lack of evidence in the literature about CYT’s safety profile in the hospitalized population.

For these reasons, safety assessment is a primary objective in the CITOSP protocol.

For CYT to be truly effective, following the adequate dosing schedule without interruption is critical. For these reasons, patient compliance and efficacy are the secondary objectives of the CITOSP protocol.

In 2021, the recall of CHAMPIX® (varenicline) from the market by Pfizer Inc. due to the presence of the impurity, N-nitroso-varenicline, a probable human carcinogen, above the acceptable daily intake limit, resulted in further shortages of CHAMPIX®, reducing therapeutic alternatives to NRT for smoking cessation (35).

CYT, such as varenicline, is a partial agonist of the nACh receptor, with a high affinity for subtype α4β2, which acts by antagonizing both the nicotinic effect and the endogenous effect of ACh. Although CYT has been known for more than 50 years as a pharmacological treatment for smoking cessation, its use is still limited. Another important advantage of CYT is that it is likely to be well accepted by hospitalized patients, who often already take multiple medications for other co-morbidities and will find it difficult to take additional medications. All these characteristics will make CYT one of the best options for helping patients quit smoking, also in a hospital context.

The CITOSP trial will be the first trial in Italy to evaluate the safety of CYT treatment for smoking cessation in a hospital context and may contribute to the knowledge of the safety profile of CYT in patients with co-morbidities.

## Data Availability

The raw data supporting the conclusions of this article will be made available by the authors, without undue reservation.
